# Transcript mapping based on dRNA-seq data

**DOI:** 10.1186/1471-2105-15-122

**Published:** 2014-04-29

**Authors:** Thorsten Bischler, Matthias Kopf, Björn Voß

**Affiliations:** 1 Genetics & Experimental Bioinformatics, Institute for Biology 3, Faculty of Biology, Albert-Ludwigs-University Freiburg, Schänzlestr. 1, 79104 Freiburg, Germany; 2 Julius-Maximilians-University Würzburg, Institute for Molecular Infection Biology, Josef-Schneider-Str. 2/D15, 97080 Würzburg, Germany

**Keywords:** RNA-seq, Differential RNA-seq, Segmentation, Transcriptional unit, Transcriptome, Transcriptional start site, Dynamic programming

## Abstract

**Background:**

RNA-seq and its variant differential RNA-seq (dRNA-seq) are today routine methods for transcriptome analysis in bacteria. While expression profiling and transcriptional start site prediction are standard tasks today, the problem of identifying transcriptional units in a genome-wide fashion is still not solved for prokaryotic systems.

**Results:**

We present RNAseg, an algorithm for the prediction of transcriptional units based on dRNA-seq data. A key feature of the algorithm is that, based on the data, it distinguishes between transcribed and un-transcribed genomic segments. Furthermore, the program provides many different predictions in a single run, which can be used to infer the significance of transcriptional units in a consensus procedure. We show the performance of our method based on a well-studied dRNA-seq data set for *Helicobacter pylori*.

**Conclusions:**

With our algorithm it is possible to identify operons and 5’- and 3’-UTRs in an automated fashion. This alleviates the need for labour intensive manual inspection and enables large-scale studies in the area of comparative transcriptomics.

## Background

The development of RNA-seq [1] has boosted research on prokaryotic transcriptomes throughout the last years. It can be used for the detection of novel transcripts, e.g. non-coding RNAs (ncRNAs), analysis of differential expression in response to environmental stimuli and others. Recently, a variant called dRNA-seq was introduced [2], which allows the transcriptome-wide mapping of transcriptional start sites (TSSs). This provides a means to reliably detect the 5’-end of transcripts. But what about the 3’-end?

The accurate determination of transcript boundaries is useful for several reasons. It enables the identification of promoter and terminator elements and, thus, UTRs which potentially carry regulatory elements. Furthermore, target prediction for ncRNAs will benefit from the knowledge of exact transcript limits and UTRs. The same holds true for expression analysis, since all reads mapping a transcript can be rigorously accounted for, which improves the precision. Not only expression levels might vary but also the transcript architecture, e.g. by differential processing or alternative TSSs. For these reasons it is of interest to have an automated procedure to detect transcript boundaries. Finally, the organization of genes into operons or transcriptional units can be easily elucidated when the genomic location of transcripts is known.

In the near past, high-density genomic tiling microarrays were the method of choice for the characterization of complete transcriptomes. For this technology a segmentation method for the hybridization signal along genomic coordinates was proposed [3]. It makes use of a structural change model (SCM) which is fitted to the array data. The goal is to partition the data into blocks with ideally uniform expression and is achieved by computing the set of segments that minimizes the sum of squared residuals. An alternative approach for the analysis of high-density oligonucleotide tiling arrays makes use of a hidden Markov model [4].

To the best of our knowledge no general method for transcript boundary estimation based on RNA-seq data is available. CUFFLINKS[5] and SCRIPTURE[6] perform best with eukaryotic mRNA-seq data, which captures polyA-tailed transcripts only, and put a focus on the detection of splice variants. Similar considerations hold true for *de-novo* transcript assemblers such as ABYSS[7] and SOAPDENOVO[8]. Prokaryotic transcripts do not have a poly-A tail and, thus, bacterial RNA-seq provides information on merely any RNA present in the cell. On the one hand, this promises to provide the full picture of a bacterial transcriptome, but on the other hand, this also increases the complexity for its analysis. Nevertheless, we set out to develop a method for transcript boundary determination based on RNA-seq data mapped to a reference genome. More precisely, we chose dRNA-seq data as the input, since it explicitly provides information on transcriptional start sites.

As a starting point we chose the SCM based segmentation algorithm from [3]. We reimplemented it in C++ and added the ability for parallel computation using openMP ( http://openmp.org). The major improvement is achieved by extending the segmentation method to make use of dRNA-seq data, especially data from libraries enriched for primary transcripts. For this, we modify the dynamic programming based optimization, such that segments satisfy certain user-defined constraints. This reduces the search space leading to improved speed and accuracy of the algorithm and further allows us to discriminate transcript from non-transcript segments. Finally, we present a method to compute consensus segments, which makes use of the fact that the algorithm intrinsically computes many results. This integrative step results in segments with improved confidence.

## Implementation

In the following we will describe the algorithm implemented in RNASEG. Due to the close relation to the algorithm developed in [3] we reuse large parts of their notation.

### dRNA-seq data

The data provided by a dRNA-seq experiment is in the simplest case a set of sequencing reads from two libraries. One library consists of sequencing reads from RNA enriched for primary transcripts, and the second is untreated. Throughout this manuscript we name the reads primary and secondary, respectively. Ideally, sequencing reads from primary libraries start at the native 5’-end of a transcript, such that the 5’-ends of primary reads represent the 5’-ends of native primary transcripts. Sequencing reads from secondary libraries start at the 5’-end of secondary transcripts. These may either be degradation or processing products or, as for the data used here, the result of a fragmentation procedure. Note that if the secondary library is treated with tobacco acid pyrophosphatase (TAP) it also contains primary transcripts, which would otherwise not be accessible for ligation due to the pyrophosphate. TAP treatment, although used in [2], is not mandatory and to make RNASEG as widely applicable as possible, we provide means to handle the effects of this treatment (see below).

We store for each position *i* in the genome the number of primary read starts *P*_*i*_, the coverage by primary reads $C_{i}^{p}$ and the coverage by secondary reads $C_{i}^{s}$.

### Structural change model segmentation

The data used for modeling consists of the number of primary read starts (*P*) and the secondary read coverage (*C*^*s*^) for each position in the genome. The coverage by secondary reads is expected to be uniformly distributed over the full-length of the transcript and, thus, RNASEG uses this data to compute the sum of squared residuals for a candidate segment. Primary read start information is used as a constraint for start positions of transcript segments and for differentiating between transcript and non-transcript segments. In the following, we recapitulate the definition from [3] and explain our extensions to incorporate dRNA-seq data.

#### Fitting the model

The SCM models the *log*_2_ normalized $C_{i}^{s}$ as a piecewise constant function of genomic coordinates as shown in (1).

(1) $$\begin{array}{l} z_{i}=\mu_{r}+\varepsilon_{i}\;\text{for}\;cp_{r}\leq i<cp_{r+1}\text{,} \end{array}$$

where *i* = 1,…,*n* is the genomic position, $z_{i}={\text{log}}_{2}(C_{i}^{s}+1)$, *cp*_1_,…,*cp*_*r*_ are parameters for segment boundaries also called *change points*, *cp*_1_ = 1 and *cp*_*R*+1_ = *n* + 1, *R* is the maximal number of segments, *μ*_*r*_ is the mean *log*_2_ normalized *C*^*s*^ for segment *r* and *ε*_*i*_ are the residuals.

The model is fitted to the data by minimizing the sum of squared residuals as shown in (2) for a predefined number of segments *R*.

(2) $$\begin{array}{l} G(cp_{1},\ldots ,cp_{R})=\overset{R}{\underset{r=1}{\sum }}\overset{cp_{r+1}-1}{\underset{i=cp_{r}}{\sum }}{\left(z_{i}-\mu_{r}\right)}^{2} \end{array}$$

The minimization which leads to the optimal set of parameters ${\tilde{\text{cp}}}_{1},\ldots ,{\tilde{\text{cp}}}_{R}$ is done by the dynamic programming (DP) algorithm described in the following section.

#### Optimization

The tool RNASEG implements an extended version of the DP algorithm available from the Bioconductor tilingArray package, which we call the original algorithm from now on. It calculates the minimal sum of squared residuals in the first step and determines the optimal set of change points during backtracing.

The original algorithm starts with computing the cost matrix *G* which is the main input for the DP procedure that finds the optimal segmentation. The cost matrix contains for each entry *G*_*i*,*k*_ the sum of squared residuals of the segment from *i* to *i* + *k* - 1. The calculation of the cost matrix for arbitrary segments would take quadratic time and space with respect to the genome size *n*. This renders the algorithm inapplicable on a genome wide scale. For our algorithm we reduce the complexity by restricting the segment length to a maximum value $\hat{k}$, resulting in a complexity of $n\times \hat{k}$ for *G*. This strategy was already chosen in the original algorithm but using a fixed, rather than a user-defined $\hat{k}$.

Just like the original algorithm, our method uses two matrices for calculating the optimal segmentation. The scoring matrix stores in each entry $mI_{i}^{\text{cp}}$ the optimal cost for the segmentation from 0 to *i* with *cp* change points. The traceback matrix contains in $mt_{i}^{\text{cp}-1}$ the index of the rightmost change point in the optimal segmentation from the start to *i* with *cp* change points. The major difference of our algorithm to the original one is, that in order to decide whether a segment is a transcript or a non-transcript segment our algorithm checks if the current segment suffices some constraints, such as enough primary reads at the segment start or a mean coverage by secondary reads above a threshold. If this is the case, the change point belongs to a transcript segment and is stored as a positive positional value in the *mt* matrix. Otherwise the change point denotes the start of a non-transcript segment and is assigned a negative positional value. A segment may even be neither a transcript nor a non-transcript, which is for example reasonable for segments with high mean coverage but without a valid TSS. In such a case, the segment is marked invalid and not further considered.

In differentiating between transcript and non-transcript segments, the algorithm allows only for transcript segments to appear one after another, or as an alternating order of transcript and non-transcript segments. The occurrence of two adjacent non-transcript segments is prevented by checking the previous change point in the *mt* matrix. This restriction is not only biologically reasonable but also results in a speed-up of the calculation.

The initialization and the recursions for the *mI* matrix are shown in (3) and (4). The algorithm computes the optimal segmentation for all different numbers of change points up to $\hat{\text{cp}}$.

(3) $$\begin{array}{l} \text{Initialization:}\\ \;{\text{mI}}_{k}^{0}=\left\{\begin{array}{ll} G_{0,k} & \text{for}\;0\leq k<\hat{k}\\ \infty & \text{for}\;\hat{k}\leq k<n \end{array}\right) \end{array}$$

(4) $$\begin{array}{l} \text{Recursion:}\\ \;{\text{mI}}_{j}^{\text{cp}}=\underset{0\leq k<k_{0}}{\text{min}}\;Z_{k}\\ \;\text{with}\;k_{0}=\left\{\begin{array}{ll} j & \text{if}j<\hat{k}\\ \hat{k} & \text{otherwise} \end{array}\right)\\ \;\text{and}\;Z_{k}=\left\{\begin{array}{ll} {\text{mI}}_{j-k-1}^{\text{cp}-1}+G_{j-k,k} & \text{val}(j-k,k,\text{cp})=\text{true}\\ \infty & \text{otherwise} \end{array}\right) \end{array}$$

where *val*(*i*,*j*,*cp*) is a function that checks if the segment from *i* to *j* for *cp* change points suffices the user defined thresholds for transcript or non-transcript segments. The individual checks are described in the following.

#### Segment constraints

In the following, (*i*,*j*) denotes a segment from position *i* to position *j* and *cp* denotes the number of change points of the current recursion.

A constraint that was already introduced above and is essential for the performance of the algorithm is the maximal segment length $\hat{k}$. In addition, the user can also impose a minimum segment length $\overset{ˇ}{k}$. The latter may be useful in cases where the dRNA-seq library preparation includes a size selection step, such that only RNAs above a certain length are analyzed. Each segment (*i*,*j*) must satisfy $\overset{ˇ}{k}\leq j-i+1\leq$$\hat{k}$.

A transcript segment (*i*,*j*) needs to start with a reasonable number of primary reads, say *t*. Therefore, the number of primary read starts at position *i* has to exceed *t* (*P*_*i*_ > *t*). This is a rather simplistic criterion, and we provide possible alternatives in the discussion, but still the complete method performs well, as will be shown later.

**Figure 1:**
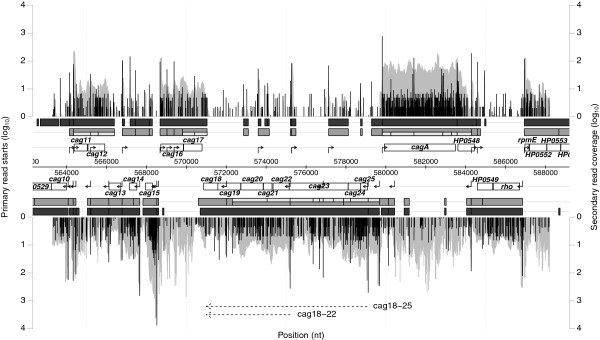
**Chromosome section 563,000 to 583,000 from** ***H. pylori* 26695.** The positional log10-normalized coverage of primary read starts (black) and secondary reads (grey) for the forward (top) and the reverse strand (bottom) is visualized. Data for the forward strand is displayed with positive values above the annotation and for the reverse strand with negative values below. Annotated genes are represented by unfilled boxes. Predicted consensus transcript segments (*T*^*s*^) are shown in grey and putative subtranscripts ( $T_{\text{sub}}^{s}$) are shown as grey inlays. Transcripts from the maximum number of change points (*T*^*m*^) are shown as dark grey boxes. TSSs and operons determined in [2] are indicated by filled and dotted arrows, respectively.

Due to differences in preparation of the secondary library, namely TAP treatment or not, the secondary libraries may also contain reads for primary transcripts. These may show up as blocks in the secondary read coverage at the start of some transcripts, e.g. *cag11* and *cag16* in Figure 1, while others (e.g. *cagA* in Figure 1) do not show this artifact. The existence of such a block artificially enlarges the cost for this segment and thus we introduce a way to skip these regions. Instead of requiring more than *t* reads at the starting position of a transcript segment, we search such a position in an area in front of a segment. The size of this search window (*w*) is a user defined parameter. In order to prevent multiple calculation of the same value an auxiliary array *N* is precomputed. Entry *N*_*i*_ stores the position *q* for which *P*_*q*_ > *t* and *q* ≤ *i*. If several *q* within *i*,…,*i*-*w* satisfy this condition, the *q* with maximum *P*_*q*_ is chosen.

For highly abundant transcripts the enrichment for primary transcripts will not be perfect, thus reads from degradation products will misleadingly show up as primary reads. The user can choose to reduce the resulting increase in potential transcript segment start points, by setting a ratio *r* (0 ≤ *r* ≤ 1) between primary read starts and primary read coverage. This changes the way in which the *N* array is computed, such that *N*_*i*_ stores the position *q* for which $P_{q}>t,q\leq i\;\text{and}\;\frac{P_{q}}{C_{q}^{p}}\geq r$ holds true. Again the *q* with maximal *P*_*q*_ is chosen within *i*,…,*i* - *w*.

A segment (*i*,*j*) is classified as a transcript segment if *i* - *N*_*i*_ ≤ *w*. This remapping of the start position is reflected in the scoring scheme by replacing $mI_{j-k-1}^{\text{cp}-1}+G_{j-k,k}$ with $mI_{N(j-k)-1}^{\text{cp}-1}+G_{j-k,k}$ in (4).

An essential feature of RNASEG is the discrimination between transcript and non-transcript segments. As described before, the *mt* matrix stores positive and negative positional values for transcript and non-transcript segments, respectively. A non-transcript segment is only allowed to follow a transcript segment, while transcript segments are not constrained, thus $(N_{i}>w\wedge mt_{i-1}^{\text{cp}-2}>0)\vee N_{i}\leq w$ must hold true.

Additional constraints can be imposed on the mean coverage *μ* of segments. For transcript segments the user can provide a minimum cut-off *a*, which needs to be exceeded by transcript segments. Vice versa, for non-transcript segments the user may provide an upper limit *u*, which must not be exceeded by non-transcript segments. We use (5) to verify this.

(5) $$\begin{array}{l} \text{vc}(i,j)=\left\{\begin{array}{rr} \text{true}, & i-N_{i}\leq w\;\wedge \mu_{\text{ij}}>a\\ \text{true}, & i-N_{i}>w\;\wedge \mu_{\text{ij}}\leq u\\ \text{false}, & \text{otherwise} \end{array}\right)\\ \;\text{with}\;\mu_{\text{ij}}=\frac{1}{j-i+1}\overset{j}{\underset{l=i}{\sum }}C_{l}^{s} \end{array}$$

Note, that we use the non-normalized $C_{i}^{s}$ here, compared to the *log*_2_ normalized values for computing the *G* matrix. We feel that this is more intuitive for the user.

For certain numbers of change points, the imposed restrictions may lead to an invalid segmentation, i.e. for a certain position *j* in the genome no *i* can be found, such that (*i*,*j*) satisfies all constraints. We mark such instances by setting $mI_{i}^{\text{cp}}=-1$ and $mt_{i}^{\text{cp}-1}=2n$. During the recursion, if a candidate segment does not satisfy $mI_{i-1}^{\text{cp}-1}\neq -1$, it is not considered a valid sub-solution and, thus, ignored.

During traceback the positions of the optimal segmentation for each number of change points are stored in the result matrix *th*. The procedure works as shown in Algorithm 1. The two if-statements check if the current trace contains a segment for which no valid segmentation could be computed during the recursion. In this case, the current backtracing is stopped and the corresponding change point number *cp* tagged invalid.

The output of the algorithm contains the transcript and non-transcript segments for each number of change points in *GFF* format. It is generated by parsing the change points stored in the *th* matrix thereby generating entries for transcript or non-transcript segments in the output file. Change point numbers which have been tagged invalid during backtracing will be ignored and will not appear in the output. For each segment the start position is the current change point *i* and the end position is located one position in front of the following change point.

### Optimal segment number and consensus segments

RNASEG computes for each number of segments, the optimal set of change points. In other words, the algorithm does not provide the overall optimal solution, but rather many solutions which are optimal by themselves, i.e. for the given number of change points. The choice of the optimal number of change points is not trivial, as has already been stated in [3]. One can use information theoretical approaches, such as the AIC (Akaike’s Information Criterion) and BIC (Bayesian Information Criterion), but the authors finally suggest an empirical estimation based on positive control regions. In our opinion, this is not satisfying and we provide two ways to cope with this.

An intrinsic property of the constraints described above is that they limit the maximal number of transcript segments $\hat{M}$. Since a non-transcript segment has to be followed by a transcript, a maximum of $2\hat{M}+1$ segments are possible. During the calculation of *N*RNASEG determines how many positions satisfy the transcript start constraints, which gives an upper bound for $\hat{M}$. If $\hat{\text{cp}}>2\hat{M}+1$RNASEG automatically lowers $\hat{\text{cp}}$ to $2\hat{M}+1$, saving computation time and memory. The maximal number of change points for which a valid segmentation could be derived depends on all constraints and is available at the end of the run. This set of segments provides the highest resolution for the given constraints. For high quality data and reasonable constraints, it likely constitutes the final result.

Our second strategy makes use of all segmentations for the different change point numbers. For similar numbers of change points, the segment sets likely share a large number of segments. It is important to note that the computation does not enforce this behavior. In order to take advantage of this information we apply a consensus strategy. This strategy focuses on transcript segments (transcripts for short) and has essentially four steps: 

• First, transcripts are collected from all numbers of change points, and their occurrence frequencies determined. We use this occurrence frequency as a proxy for the quality of the prediction.

• Second, internal TSSs may split a long transcript into two or more short transcripts. Hence, transcripts that together correspond to a longer transcript, i.e., sub-transcripts, are chained and their occurrence frequencies added to the long version.

• Third, transcripts from different numbers of change points may differ only by a few positions. Thus, we merge transcripts that overlap to 99% or more. For this, we keep the more frequent variant and sum up the occurrence frequencies.

• Fourth, for competing (partially overlapping) transcripts we retain the one with higher cumulated occurrence frequency, as this is supported by a larger number of individual segmentations.

As a result, the segment sets for the various change point numbers are merged and provided in a single output file in GFF format, ready for visualization in popular genome browsers. Furthermore, transcript segments are augmented by their occurrence frequencies among all change point numbers, which allows to infer the significance of the actual transcript. As a byproduct this script allows to merge results for the different strands as well as of several partial analyses of adjacent, possibly overlapping, genomic regions. Thus, it is easy to split the analysis of a complete genome into small, overlapping pieces (say 100 kb), do the segmentation piecewise, and merge the individual results. This decreases overall runtime, since for shorter sequences $\hat{\text{cp}}$ can also be reduced.

## Results

We applied RNASEG to the data from [2] for *Helicobacter pylori* 26695. The individual steps are described in the following.

### Data

We downloaded all data for the experiments SRX014058, SRX014056, SRX014054, SRX014051, SRX014034, SRX014033, SRX014031, SRX014018, SRX014013-17 from the NCBI Sequence Read Archive (SRA). The samples SRX014013-17 represent Solexa sequencing results of untreated RNA, while the other samples correspond to 454 sequences from primary enriched libraries. In total approx. 2 million primary and 83 million secondary reads were obtained. All reads were polyA trimmed at the 3’-end and 454 reads were additionally subjected to a 5’ adapter clipping (fixed length clipping using 28 bases).

### Read mapping and input file generation

We used SEGEMEHL[9] to map the reads to the genome, requiring a minimum accuracy of 85% and utilizing the co-optimal matching strategy.

In the following a positional coverage file for each strand was generated where the primary read starts and coverage were calculated as the maximum of the primary libraries. The secondary coverage is the mean of the secondary libraries. In both cases the values were normalized beforehand via the number of mapped reads for the respective library. In order to be suitable as input for RNASEG the data was arranged in tab-delimited format as follows: Each row has four values corresponding to primary read starts, primary coverage, secondary read starts and secondary coverage. The genomic position is not represented explicitly, but given implicitly by the position of the row in the file. Thus, values in row 1 correspond to position 1 in the genome, row 2 to position 2, and so on. We selected this format since it can be directly visualized by the Artemis genome browser [10] as a user graph. Together with the output of RNASEG in GFF format this allows a simple and fast visualization of the experimental data together with the prediction. The RNASEG distribution provides a python script to convert mapping files in SAM format to the described format.

### Application

In order to speed up the computation, we analyzed the 1,667,867 nt long genome in 17 parts of 103,112 nt length where adjacent parts overlap by 10,000 nt. RNASEG was applied with the following constraints: primary read start threshold *t* = 10, min./max. segment length $\overset{ˇ}{k}=50,\hat{k}=10,000$, maximal no. of change points $\hat{\text{cp}}=1,000$, transcript mean coverage cut-off *a* = 0.5, non-transcript max. mean coverage *u* = 0.5, and read start ratio *r* = 0.5. These settings were derived by analyzing small sample regions. The results were combined using our consensus strategy described above and transcripts with an occurrence frequency below $\frac{1}{4}$ discarded. In total, 1,696 transcripts and 2,147 sub-transcripts were predicted. We also extracted the transcripts for the maximal number of change points for each analyzed part and joined those that meet without a gap within an annotated gene, resulting in 2,150 transcripts. We term transcripts and sub-transcripts derived by summarizing *T*^*s*^ and $T_{\text{sub}}^{s}$ and those from the maximum change points *T*^*m*^.

Figure 1 shows the results for section 563,000 to 583,000. This region comprises the *cag* pathogenicity island, which was also described in detail in [2]. Overall, the coverage plots give an impression about the complexity of the data. *T*^*m*^ segments (dark grey boxes) or blocks of adjacent *T*^*s*^ segments (grey boxes) nicely reflect the genomic organization in this region. The two alternative operons (cag18-25 and cag18-22) suggested in [2] can be confirmed when taking into account the $T_{\text{sub}}^{s}$ (light grey inlays). In total, 39 and 57 *T*^*m*^ and $T^{s}/T_{\text{sub}}^{s}$ segments, respectively, were predicted from which 23 and 31, respectively, correspond to the 31 manually selected TSSs from [2]. Among them the acid induced internal TSS in *cag23*.

**Figure 2:**
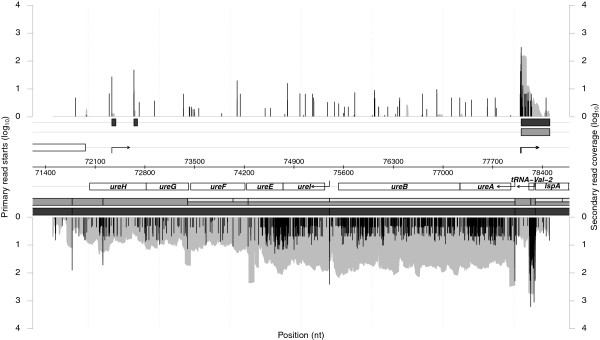
**Chromosome section 71,500 to 78,500** ***H. pylori* 26695.** Data is arranged in the same way as in Figure 1.

The second example in Figure 2 shows the region from position 71,500 to 78,500 and comprises the urease operon (*ureA-ureH*). Again our analysis confirms the operon organization *ureAB* and *ureIEFGH* presented in [2], especially for the *T*^*m*^ segments that show perfect agreement. Sharma *et al.*[2] manually derived operon structures by taking into account the operon prediction from the DOOR [11] database. We extracted those operons described as new, confirmed, alternative or extended and neglected those termed ambiguous. This resulted in 332 operons, some of which had two or more proposed alternatives, which we used as our reference set. For the predicted segments we defined operons as those genes overlapping the same transcript segment. For each operon from the reference set we looked for the closest predicted segment, where the distance was defined by the number of different genes. 234 out of 332 operons were equal, 41 differed by one gene, 22 by two, 19 by more than two, and 16 operons were not predicted at all. In addition, 23 new operons were predicted by RNASEG.

#### Simulated data

To test the influence of the parameters on the performance of RNASEG we used simulated data. Since no dRNA-seq read simulator is currently available we modified an RNA-seq read simulator, namely RNASeqReadSimulator [12] as follows: First, reads are Poisson distributed over the transcript and, second, a user-defined fraction between 0–100% is assigned to the first position, which simulates the enrichment of primary transcripts in the primary dRNA-seq library. The simulation scripts are part of the RNASEG distribution. We simulated a primary (50% primary reads) and a secondary library with approx. 50 million reads each, and applied RNASEG with varying values for the primary read start cut-off *P*, the mean transcript coverage cut-off *a* and the mean nontranscript coverage cut-off *u*. We used the F-Measure and the fraction of recovered reads from the secondary library to assess the performance. The results are summarized in Table 1. RNASEG achieves a maximal F-Measure of 0.93, but interestingly the fraction of recovered sequencing data in transcript segments reaches 100*%*. The reason for this is that the section we analyzed contains genes for which very few or even no reads were simulated. If we would count these as true negatives the F-measure would be 1.0. The F-measure drops to 0.67 for high values of *a* and *u*, but still the fraction of recovered sequencing data stays high (above 97%). This shows, that mainly genes with low read numbers are affected.

**Table 1:** RNASEG** results on simulated data**

		***a***	**F-measure**				**Recovered sequencing data [%]**			
	***u***		**1**	**10**	**100**	**1,000**	**1**	**10**	**100**	**1,000**
*t* = 1 (170 h)		1	0.93	0.93	0.93	0.93	100.00	100.00	99.95	97.92
		10	-	0.93	0.92	0.93	-	100.00	99.96	98.10
		100	-	-	0.86	0.86	-	-	99.91	99.12
		1,000	-	-	-	0.67	-	-	-	97.73
*t* = 100 (156 h)		1	0.93	0.93	0.93	0.92	100.00	100.00	99.96	98.63
		10	-	0.93	0.92	0.92	-	100.00	99.96	98.10
		100	-	-	0.86	0.86	-	-	99.91	99.12
		1,000	-	-	-	0.67	-	-		97.73
*t* = 1,000 (110 h)		1	0.87	0.86	0.83	0.81	100.00	99.98	99.83	97.76
		10	-	0.86	0.83	0.81	-	99.98	99.83	97.76
		100	-	-	0.82	0.80	-	-	99.81	97.76
		1,000	-	-	-	0.67	-	-	-	97.75

F-measure and fraction of recovered sequencing data from the secondary library (in %) for simulated data. RNASEG was applied to the forward strand of region 684,676 to 987,046 of the *H. pylori* genome. We set $\hat{\text{k}}$ = 20,000, $\hat{\text{cp}}$ = 1,000, *w* = 100, and varying values for parameters *t*, *a* and *u*. We define: True positives (TP) are genes that are part of a transcript segment, true negatives (TN) are intergenic regions that are not part of a transcript segment, false positives (FP) are intergenic regions that are part of a transcript segment and false negatives (FN) are genes that are not part of a transcript segment. ‘-’ indicates parameter combinations that have not been tested because they are not sensible. Numbers in brackets below *t* correspond to the average runtime in CPU hours for all simulations with this value of *t*.

### Performance

As mentioned before, we restrict the computation allowing only for a maximum segment length $\hat{k}$. Nevertheless the algorithm is still computationally demanding when applied on a genomic scale. Memory consumption can be estimated as follows. The dominating elements are the matrices *G*, *mI* and *mt* and their sum accounts for over 99% of the memory consumption. Given that each value is stored in 8 bytes (double precision float) the *G* and *mI* matrices need $8n\hat{k}$ and $8n\hat{\text{cp}}$ bytes of memory, respectively. The *mt* matrix stores integer values needing only 4 bytes and thus its memory consumption is $4n\hat{\text{cp}}$, which is half that of *mI*. In total the memory consumption can be estimated with the equation $8n(\hat{k}+1.5\hat{\text{cp}})$ bytes.

The runtime scales linearly with *n*, $\hat{k}$ and $\hat{\text{cp}}$ each. For each *cp* we compute optimal segmentations for each 0 < *i* ≤ *n*. Interestingly, the computation for each *i* depends solely on results from the previous change point numbers, thus allowing for parallel computation over all *i*. For this we make use of openMP and the runtime scales nearly reciprocal-linear with the number of threads. Computation of our presented results took roughly 8 hours, using 30 CPU cores (AMD Opteron 6282 SE) and a maximum of 12 GB of memory.

RNASEG also checks the values of $\hat{k}$ and $\hat{\text{cp}}$ for plausibility. During the computation of the array *N*, the algorithm counts the number of possible starts *π* and determines the largest gap between two adjacent starts *δ*. If $\hat{\text{cp}}>2\pi +1$ it is reduced to 2*π* + 1 and if $\hat{k}<\frac{\delta }{2}$ it is increased to $\frac{2\delta }{3}$. The conservative increase of $\hat{k}$ is a compromise between increased runtime and the chance to get a valid segmentation. Note that a gap between putative TSSs may be overcome by two segments, one transcript and one non-transcript segments. Thus, in theory gaps of size $2\hat{k}$ may be segmented correctly.

The parameter *t* controls the number of putative transcript starts, and thus effects the values of $\hat{k}$ and $\hat{\text{cp}}$. As a rule of thumb, the higher *t*, the lower $\hat{\text{cp}}$ and the higher $\hat{k}$. For our simulated data this effect is reflected by decreased runtimes for higher values of *t*. Memory consumption was more or less constant at 46 Gb because the automatically adjusted $\hat{\text{cp}}$ numbers (273, 255 and 171 for t = 1, t = 100 and t = 1,000 respectively) were on a relatively low level compared to $\hat{k}$ and *n* (20,000 and 302,371, respectively), which dominate memory usage.

## Discussion

Using the SCM approach we developed a tool, namely RNASEG, for the mapping of 5’ and 3’ transcript boundaries based on dRNA-seq data. Previous dRNA-seq based studies on bacteria [2,13] mainly made use of primary libraries to identify different classes of TSSs, but neglected 3’-ends. These are of special interest for *cis*-antisense or *trans*-acting sRNAs which lack a coding sequence to determine their approximate range in the genome. Our results show that, despite the partly noisy data, RNASEG performs well and can be used to infer transcriptional units from dRNA-seq experiments. Compared with a manually curated operon prediction, our method reconstructs 70% of the known operons and misses many others by only a few genes. This failure can be mainly attributed to the presence of internal TSSs, which result in the prediction of several adjacent transcripts rather than a long one. Furthermore, these alternative transcripts might constitute interesting operon variants. Availability of more robust data together with algorithmic improvements, as described below, will likely yield even better results.

We expect predicted 5’-ends of transcripts to be more accurate than their 3’-ends for two reasons. First, primary libraries within a dRNA-seq experiment provide distinct information on the 5’-ends of transcripts and we do not have such data for 3’-ends. Second, transcription termination is not as specific as transcription initiation. Especially, Rho-independent termination does not lead to defined 3’-ends since it is a dynamic process guided by the RNA itself ( [14], Review). The thermodynamic characteristics of the terminator hairpin and the successive U-tail heavily influence termination efficiency [15] and read-through is a common phenomenon.

A recent study of the transcriptome of the cyanobacterium *Synechococcus elongatus* PCC 7942 [16] also applies the SCM approach for the identification of non-coding transcripts. Here, for non-coding transcripts the segmentation is applied strand-specific to 15 kb intervals with 5 kb overlaps and 30 change points. Segments with a mean coverage below two reads per nucleotide are classified as non-transcribed regions and removed together with segments overlapping previously defined transcripts for annotated genes. For the remaining segments the 5’- and 3’-end are adjusted using a statistical approach, which models the positional drop in read coverage by a binomial test. By design, this test compares positions 1 and 2 nt apart, thus it is susceptible to noise, especially for low coverage transcripts.

The two widely used tools for transcript assembly in eukaryotic studies, CUFFLINKS[17] and SCRIPTURE[6], are tailored to detect transcript isoforms. They are designed for RNA-seq of mRNAs, which makes use of the polyA-tail for cDNA synthesis, and perform best with paired-end data. In contrast to SCRIPTURE, CUFFLINKS can be applied to non-paired-end data. Although the authors do not recommend CUFFLINKS for the analysis of bacteria [18], we have applied it to our data with default settings and got no reasonable results (data not shown). We think, that the problems mainly originate from the data. RNA-seq and also dRNA-seq data from bacterial transcriptomes harbour much more noise than polyA-guided RNA-seq data from eukaryotes. Furthermore, our data does not provide paired-end information.

Currently, TSS identification within RNASEG is mainly based on primary read starts that have to exceed a given threshold. False positives may be ruled out by the fact that a TSS has to be connected to a region satisfying the transcript segment constraints. For low abundant transcripts, a constant threshold may be too simplistic and we may choose a more sophisticated method in a future version. Here, the approach used in [19] seems promising to us, since it explicitly makes use of the two libraries provided by a dRNA-seq experiment. Roughly speaking, the read start counts of both the libraries are modeled by a Poisson distribution and the difference of these distributions (which follows a Skellam distribution) is used to compute p-values, based on which significant TSSs are identified.

The sequencing costs will drop substantially within the next years, thus more sophisticated data sets will become affordable. Especially, data for different growth conditions and with biological replicates will become standard. RNASEG can be easily extended to make use of these. For example, replicate data will contribute equally to the sum of squared residuals, as it is already implemented in [3]. Different growth conditions may be used in such a fashion, that the change of the primary starts at the mapped transcript start should be similar to the change of the mean secondary coverage of the complete transcript. The relation of these two measures is likely not linear and, thus, needs to be carefully analyzed.

Runtime and memory consumption are quite large for the current version of RNASEG. We have several ideas how to improve on this. One solution would be to decrease the resolution. At the moment we work with single-nucleotide resolution, but switching to, e.g., 10 nt resolution would decrease runtime and memory consumption nearly by a factor of 100. Of course, we would loose accuracy but mainly for the 3’-ends since the mapping of segment starts to positions with a reasonable number of primary starts can still be done with single nucleotide precision. Memory consumption would benefit in the same way from the reduced resolution.

## Conclusions

With RNASEG we provide the first method for the prediction of transcription units tailored for dRNA-seq data. It will help in whole-transcriptome characterization and in the identification of operon structures and 5’- and 3’-UTRs. The latter are important regions in post-transcriptional gene regulation by ncRNAs and, thus, the results will improve subsequent studies, such as ncRNA target prediction or the identification of *cis-*regulatory elements and transcription termination signals.

## Availability and requirements

**Project name:** RNASEG;

**Project home page:**http://www.comptrans.uni-freiburg.de/Software/RNAseg;

**Operating system(s):** Platform independent;

**Programming language:** C++;

**Other requirements:** Boost libraries >= 1.23;

**License:** GNU GPLv2

## Competing interests

The authors declare that they have no competing interests.

## Authors’ contributions

TB implemented the software and drafted the manuscript, MK analysed the data, BV conceived of, designed and coordinated the study and wrote the manuscript. All authors read and approved the final manuscript.
